# Regulation of the Immune Response by the Inflammatory Metabolic Microenvironment in the Context of Allotransplantation

**DOI:** 10.3389/fimmu.2018.01465

**Published:** 2018-06-25

**Authors:** Nicolas Degauque, Carole Brosseau, Sophie Brouard

**Affiliations:** ^1^CRTI UMR 1064, INSERM, Université de Nantes, Nantes, France; ^2^Institut de Transplantation Urologie Néphrologie (ITUN), CHU Nantes, Nantes, France

**Keywords:** allotransplantation, immunometabolism, T lymphocytes, lactic acid, acetate, B cells, adenosine triphosphatases, inflammation

## Abstract

Antigen challenge induced by allotransplantation results in the activation of T and B cells, followed by their differentiation and proliferation to mount an effective immune response. Metabolic fitness has been shown to be crucial for supporting the major shift from quiescent to active immune cells and for tuning the immune response. Metabolic reprogramming includes regulation of the balance between glycolysis and mitochondrial respiration processes. Recent research has shed new light on the functions served by the end products of metabolism such as lactate, acetate, and ATP. At enhanced local concentrations, these metabolites have complex effects in which they not only induce T and B cell responses, cell mobility, and cytokine secretion but also favor the resolution of inflammation by promoting regulatory functions. Such mechanisms are instrumental in the context of the immune response in transplantation, not only to protect the graft and/or eliminate cells targeting it but also to maintain cell homeostasis *per se*. Metabolic adaptation thus plays an instrumental role on the outcome of the cellular and humoral responses. This, of course, raises the possibility of drugs that would interfere in these metabolic pathways to control the immune response but also highlights the risk that some drugs may perturb this metabolism and cell homeostasis and be deleterious for graft outcome. This review focuses on how metabolic alterations of the local immune microenvironment regulate the immune response and the impact of metabolic manipulation in allotransplantation.

## Introduction

The research devoted to immunometabolism over the last decade has highlighted the cross talk between immune networks and metabolic pathways (1) to adjust the energetic machinery of lymphocyte and to fulfill the needs of an effective immune response. Whereas rapid replication of naïve and memory T and B cells results from the integration of antigen-driven stimuli, costimulatory molecules, and cytokine pathways, the effector immune response also results in the differentiation of T and B lymphocytes. B cells will ultimately differentiate into antibody (Ab)-producing plasma cells, whereas the cytokine secretion of various CD4 T helper cells (T_h_1, T_h_2, T_h_17, and T_h_22) will orchestrate the immune response. The combined functions of cytokine secretion and cytotoxic action of CD8 T cells will elicit the elimination of target cells and protect the body from intracellular pathogens and tumors. A major contribution of immunometabolism research has been to highlight how metabolic fitness is crucial to support this shift from quiescent to active immune cells and how the survival of such long-term naïve and memory lymphocytes is fine-tuned by their metabolism. It is thus not surprising that transplant immunologists exhibit a growing interest in the field of immunometabolism. This is particularly true in fields of transplantation where the current suppressive drugs impact both immunity and metabolism. Understanding how the immune system adapts to the chronic stimulation triggered by allogeneic transplant and how metabolism interacts with treatment will likely improve the standard of care for patients and increase the survival of graft recipients. This review focuses on how metabolic alterations of the local immune microenvironment regulate the immune response and what implications these effects hold for allotransplantation.

## Cross Talk Between Metabolism and T Cell-Based Immunity

The ability of T cells to adapt their metabolic status in response to change in the external microenvironment has attracted increasing interest in recent years. The integration of metabolic signals has a profound impact on specific immune cell responses. Quiescent and activated T cells rely on different metabolic pathways to sustain their energetic needs. Oxidative phosphorylation (OXPHOS) is used mainly in the quiescent state by naïve or memory T cells. By contrast, the proliferation and effector functions of activated T cells upregulate their glycolytic flux to support the biosynthesis of proteins, nucleic acids, and lipids.

## Balance of Glucose and Oxphos Metabolism in T Cell Response

A hallmark of T cell activation is the switch from OXPHOS to aerobic glycolysis. After uptake by the glucose transporter (GLUT), glucose molecules are converted into glucose-6-phosphate and will later (i) fuel the pentose phosphate pathway (PPP) to provide ribose-5-phosphate, a precursor for nucleotide synthesis; (ii) generate reducing equivalents (NADPH) for fatty acid synthesis; and (iii) fuel the tricarboxylic acid (TCA) cycle through the uptake of pyruvate by mitochondria. Glycolysis yields lactate as an end product and results in the net production of two ATP per glucose.

The metabolic conversion of T cell metabolism from OXPHOS to aerobic glycolysis is a pre-requisite for most but not all T cell function. Indeed, the activation of naïve CD4 T cells can occur when aerobic glycolysis is inhibited, rather than in the context of OXPHOS inhibition (2). The ATP synthase inhibitor oligomycin is sufficient to prevent the activation and proliferation of naïve CD4 T cells. Mitochondrial ATP from OXPHOS and not aerobic glycolysis is sufficient to support T cell proliferation as naïve CD4 T cells proliferate when activated in medium supplemented only with galactose. Upon activation, T cells can use either aerobic glycolysis or OXPHOS to support their proliferation, as shown by the proliferation of activated CD4 T cells despite the administration of oligomycin 2 days after activation. Nevertheless, aerobic glycolysis is needed to support cytokine production, as T cells cultured in galactose-supplemented medium have severe defects in IFN-γ and IL-2 production. The defective cytokine production is likely due to a block in translation rather than transcription as the expression of IFN-γ and IL-2 transcripts or the protein expression of transcription factor T-bet are similar in glucose- and galactose-cultured cells. Collectively, these data demonstrate that cytokine mRNA translation is regulated by aerobic glycolysis.

The glycolytic enzyme GAPDH not only has metabolic functions (3) but also acts as an mRNA-binding protein that regulates mRNA translation (4). GAPDH is able to bind to adenylate/uridylate-rich elements in the 3′ UTRs of IFN-γ and IL-2 mRNAs (5). CD4 T cells cultured in galactose-supplemented medium show a 10-fold increase in GAPDH-associated IFN-γ transcripts compared with cells cultured in glucose-supplemented medium (2). Therefore, aerobic glycolysis promotes effector cytokine production by distracting GAPDH from binding to cytokine transcripts. For instance, in a mouse model of infection with *Listeria monocytogenes*, the CD4 T cells expressing the highest amounts of GAPDH secrete low amounts of IFN-γ (2). Activated T cells increase also the expression of lactate dehydrogenase A (LDHA) to support aerobic glycolysis by relieving the burden on mitochondria that burn acetyl-CoA to generate ATP (6) and by regenerating NAD^+^ consumed by GAPDH during glycolysis. For instance, LDHA-deficient CD4 T cells exhibit a 30% decrease in glucose consumption compared with WT cells (6). LDHA deficiency protects mice from lethal autoinflammatory disease induced by stable expression of IFN-γ in Yeti/Yeti mice (6, 7). LDHA helps maintain high concentrations of acetyl-CoA that can be readily used as a substrate for histone acetyltransferases for epigenetic regulation of target genes including IFN-γ. Thereby, aerobic glycolysis promotes effector T cell differentiation through an epigenetic mechanism.

In addition to its key role in eliciting T cell activation, glycolysis has been shown to control the inductive and suppressive functions of human regulatory T cells by modulating the expression of *FOXP3* splicing variants containing exon 2 (8). Suboptimal stimulation of T_conv_ cells leads to the generation of highly suppressive human iT_reg_ cells. These cells are characterized by a high-glycolytic rate and constitute the metabolically active fraction of T_conv_ cells. Inhibition of glycolysis by 2-DG (2-deoxy-d-glucose) blunts the IL-2–IL-2R–STAT5 signaling pathway and consequently limits the generation of human iT_reg_ cells and their suppressive functions (8). By contrast, the inhibition of fatty acid oxidation (FAO) by etomoxir (Etx) has the opposite effect and enhances the generation of iT_reg_ cells (8). It is interesting to observe that T_conv_ cells from patients with RRMS or T1D displayed an impairment of glycolysis despite showing no defect regarding proliferation after CD3/CD28 stimulation. Moreover, iT_reg_ cells generated from the T_conv_ cells of RRMS patients have diminished suppressive function compared with similar cells from age- and gender-matched healthy control individuals. The reduced suppressive function of iT_reg_ cells from RRMS patients is associated with lower expression of CTLA-4, PD-1, Foxp3-E2, and CD71 (8).

Collectively, these reports highlight the complexity of the regulation of T cell immune response by aerobic glycolysis; this metabolic pathway is critical to mount an efficient T cell response, as well as to generate and sustain the suppressive function of regulatory T cells by regulating the expression of Foxp3-E2, which is necessary for the suppressive function of human iT_reg_ cells.

## Lipid Metabolism and Its Impact on T Cell Response

Lipids are key structural components of the cell membrane, and T cells double their lipid content in preparation for each round of cell division. In addition to their key structural properties, lipids are also used to generate energy through the process of β-oxidation. Interestingly, lymph nodes are surrounded by adipose tissue, and thereby facilitate the access of the immune system to lipid sources (9). *In vivo* LPS injection results in rapid but transient spontaneous lipolysis in the adipocytes surrounding the popliteal lymph nodes, whereas the response of adipocytes in other anatomical locations is limited (10). Similarly, there is a change in adipose tissue distribution, with fat depots surrounding lymphoid tissue in patients with chronic diseases such as Crohn’s disease and in long-term treated HIV patients (11).

Enhanced mitochondrial FAO constitutes one of the hallmarks of metabolic reprogramming required for the generation of memory CD8 T cells (12). It has been shown that, upon activation, memory CD8 T cells do not increase their uptake of external long-chain fatty acids but rather synthesize fatty acids *de novo* to support FAO (13). The lysosomal hydrolase lysosomal acid lipase is more highly expressed in *in vitro*-induced memory CD8 T cells than in effector CD8 cells and supports the generation of free fatty acid (FA) and cholesterol in the lysosomes (13). Fatty acid metabolism also impacts the differentiation of CD4 T cells. Inhibition of fatty acid synthesis by selective inhibition of acetyl-CoA carboxylase 1 (ACC1) favors the generation of human and mouse regulatory Foxp3^+^ T_reg_ cells and restrains the formation of pro-inflammatory Th17 cells (14). Whereas T_reg_ cells take up exogenous fatty acids to produce phospholipids for the cell membrane, Th17 cells rely on ACC1-mediated *de novo* fatty acid synthesis. As a consequence, provision of soraphen A, a specific inhibitor of ACC, attenuates the *in vivo* development of EAE by shifting the T_h_17/T_reg_ balance toward a pro-tolerogenic profile. These data indicate that targeting fatty acid synthesis may be an option for immunomodulation.

## Lactate, More than a Waste Product of Cellular Metabolism

For decades, lactate has been considered a waste product of cellular metabolism. Production of lactate occurs as a consequence of high-glycolytic flux in dividing cells or under hypoxic conditions. Two forms of lactate are present, either at higher pH as the ion salt (i.e., sodium lactate) or at low pH as the acid in its undissociated form (i.e., lactic acid). Thus, the negatively charged biologically active form (i.e., sodium lactate) represents the main form under physiological conditions (pH 7.2). The organic molecules that fuel mitochondrial metabolism *in vivo* are not fully understood, and it has been demonstrated recently that lactate fuels mitochondria in both normal and cancerous tissue (15).

Aerobic glycolysis was initially proposed by Otto Warburg in the 1920s after the observation of a high amount of lactate production by tumor cells *ex vivo*. This original observation led to the assumption that mitochondrial metabolism has a minor role in the production of macromolecules. Infusion of ^13^C-glucose or ^13^C-lactate tracer into patients with lung cancer has demonstrated this assumption to be wrong. Indeed, enhanced glycolysis is associated with fueling of the TCA cycle by lactate (15). The in and out transport of lactate is mediated by the lactate dehydrogenases LDHA and LDHB and by the proton-coupled monocarboxylate transporters (MCTs 1–4), members of the solute carrier 16a family of 12-transmembrane-domain, proton-linked monocarboxylic acid symporters (SLC16A1/7/8/3). MCTs bidirectionally cotransport H^+^ ions and lactate anions, depending on their respective concentration gradients. Continuous lactic acid efflux is inhibited by an excess of external lactic acid, and thereby hampers T cell metabolism. Recently, Hui et al. demonstrated that lactate is, by contrast, the predominant fuel for mitochondrial metabolism (15). Lactate shows a 2.5-fold higher circulatory turnover flux than glucose in fasted mice, although glucose was previously considered the predominant circulating carbon source. Indeed, lactate has the highest circulatory turnover flux of any metabolite, exceeding that of glucose by 2.5-fold in fasting mice and 1.1-fold in fed mice (15). Thus, in all tissues except the brain, circulating lactate is the main contributor to tissue TCA metabolism. The Cori cycle postulates that lactate is produced upon anaerobic glycolysis in the muscle and later converted to glucose in the liver before being metabolized back to lactate after its return to the muscles. The potential for lactate to transfer carbon between tissues has already been shown (16, 17), leading to the concept that glycolysis and OXPHOS are tightly linked pathways and opposing processes, as the product of glycolysis provides the substrate for OXPHOS (16). The use of a ^13^C tracker recently provided evidence to support this concept by demonstrating that glucose feeds TCA metabolism mainly through circulating lactate (15).

### Regulation of T Cell Motility and Cytokine Secretion by Lactate

Glycolysis results in the production of pyruvate, which is reduced to lactate by LDHA/B, a process coupled with the conversion of NAD^+^ to NADH. Lactate secretion maintains the intracellular pH by eliminating protons through the MCTs and therefore allows the persistence of the glycolysis rate. Increasing the amount of lactate in the external milieu inhibits glucose consumption by reversing the flux of lactate and can thereby inhibit T cell function. It has been shown, for instance, that lactate tightly regulates the motility and migration of CD4 and CD8 T cells (18). Lactic acid but not sodium lactate inhibits CD8 T cell migration (18). The progressive acidification of the medium induced by the provision of lactic acid does not account for the reduction of cellular motility of CD8 T cells. By contrast, sodium lactate and not lactic acid inhibits the migration of CD4 T cells by interfering with glycolysis and favors the production of the pro-inflammatory cytokine IL-17 but not IFN-γ. The modulation of T cell functions depends on the expression of specific lactate transporters, namely, the lactic acid transporter Slc16a1 on CD8 T cells and the sodium lactate transporter Slc5a12 on CD4 T cells. Selective blockade of Slc16a1 and Slc5a12 reverses the blockade of transmigration of CD8 and CD4 T cells, respectively. Moreover, the selective blockade of Slc5a12 not only prevents T cell migration to the inflammatory site but also blocks the secretion of IL-17. Sodium lactate induces a decrease in extracellular acidification rate (ECAR) and in glucose uptake, and thereby blunts the glycolytic flux of CD4 T cells. Finally, a decrease in chemotaxis, as measured by an *in vitro* assay or *in vivo* in a model of T cell recruitment to the peritoneum, is observed after the direct or indirect inhibition of glycolysis with the glucose analog 2-DG or the mTOR inhibitor rapamycin. Therefore, after their migration into inflammatory sites such as the synovial joints of RA patients, T cells sense the local concentration of lactate and become trapped at the site. Despite the reduced CTL function of CD8 T cells, a chronic local inflammatory environment is sustained through the increased production of pro-inflammatory cytokines. The differential expression of lactate transporter by CD4 and CD8 T cells raises the question of how the nature of the inflammatory exudate (i.e., more lactic acid versus sodium lactate) regulates the differential distribution of T cells.

A key feature of inflammatory sites and tumors is the enhanced glycolysis resulting in the accumulation of lactic acid. Sodium lactate does not impact the proliferation of CTL CD8 induced by antigen-specific or mitogenic stimulation, whereas lactic acid can suppress their proliferation (19). Similarly, the production of IL-2 and IFN-γ by CTL is abolished by lactic acid at a concentration of 20 mM, whereas sodium lactate or acidification alone has no impact. The inhibition of CTL function by lactic acid is reversible by the removal of lactic acid. Lactate serum concentrations and tumor burden in cancer patients are positively correlated, and lactic acid but not the mere acidification of the environment inhibits key functions of CTL. The inhibitory effect of lactic acid on cytotoxic activity is also observed in NK cells and associated with reduced expression of perforin and granzyme (20).

**Figure 1:**
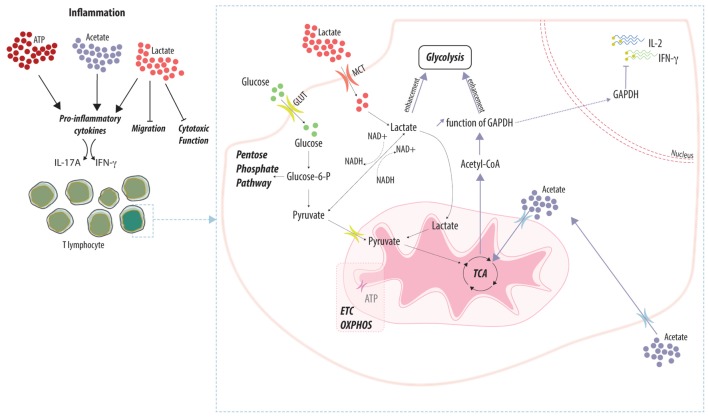
Adaptation of T cell function to the local metabolic environment. Local concentrations of metabolic by-products (e.g., ATP, acetate, and lactate) differentially impact the function of T lymphocytes. Accumulation of metabolites favors the effector function by enhancing the secretion of pro-inflammatory cytokines while favoring the retention of T cells within the inflamed tissue. Lactate and acetate are actively imported into T cells and fuel the main metabolic pathways. Enhancement of the glycolytic rate prevents the inhibitory effect of the glycolytic enzyme GAPDH on the translation of pro-inflammatory cytokines.

In short, lactate acts as a complex immunomodulatory molecule to control T cell effector functions during inflammation and to favor the retention of activated CD4 T cells (Figure 1).

## Impact of Systemic Metabolic Alterations on Immune Cell Function

Sensor molecules able to detect danger are necessary to mobilize an efficient immune response. Acetate and extracellular ATP (eATP) are two such molecules, and their metabolic pathways are involved in the regulation of inflammation.

### Regulation of T Cell Response by Acetate Levels

It has become evident that lymphocyte T cells influence homeostasis and integrate environmental signs of danger (21). For instance, a systemic bacterial infection induces within hours an increase in acetate serum levels, and high acetate concentrations are needed to mount an optimal memory CD8 T cell response *in vitro* and *in vivo* (22). The primary production site of acetate released in the circulation is the liver (23). Hydrolysis of acetyl-CoA and release of acetate into the extracellular space can occur in other tissues under catabolic and metabolic stress conditions such as starvation or diabetes (24). Under physiological and normoxic conditions, the major source of cytosolic acetyl-CoA derives from the production of mitochondrial acetyl-CoA during glycolysis or β-oxidation (25). Mitochondrial acetyl-CoA is metabolized within the TCA to yield NADH, the main substrate for ATP synthesis *via* OXPHOS (26). Acetyl-CoA is also a central metabolite intermediate for lipid synthesis, and acetylation (provision of acetyl groups to the N-terminal residue of a protein) is one of the major post-translational protein modifications regulating the stability, localization, and function of proteins (25). The cellular function of metabolic enzymes is tightly regulated at the post-translational level through their acetylation (27). The concentration of acetate in the sera of mice infected with *L. monocytogenes, Salmonella typhimurium*, or *Escherichia coli* rises transiently within few hours after systemic infection (4–48 h) (22). Provision of acetate during the memory induction phase of *in vitro* differentiation of memory OT-I T cells results in increased and rapid secretion of IFN-γ upon rechallenge. Acetate has no impact on the expression of phenotypic markers or chemokine receptors. Hallmarks of memory T cells include the ability to switch rapidly to glycolysis to support IFN-γ production (28) and an increase in spare respiratory capacity (SRC) (29). Acetate exposure increases the glycolytic reserve of memory CD8 T cells by twofold in addition to increase the SRC (22). The increase in IFN-γ production upon acetate exposure is reversible, as the secretion of IFN-γ returns to the basal level when acetate is removed from previously acetate-exposed memory OT-I cells.

Therefore, a systemic increase of acetate during an inflammatory response is integrated by the immune system to favor the generation of efficient memory CD8 T cells, another new example of connection between the modulation of the immune response and systemic metabolism (Figure 1). The alteration of the tissue microenvironment by regulating the level of acetate will dictate the fate of T cells and is likely to represent an attractive way to control allo- and autoimmune response.

### eATP: A Key Factor in Inflammation and Immune Responses

Intracellular adenosine triphosphate is well known as the energy source driving cell survival, proliferation, and metabolic function (30). However, under tissue stress such as hypoxia, apoptosis, necrosis, or inflammation, ATP can be released from cells into the extracellular environment (31–33). Two mechanisms are involved in the release of ATP into the extracellular space: (i) passive ATP release by necrotic cells by loss of cell membrane integrity and (ii) active ATP release through transporters/channels or exocytosis (34). This ATP release through transporters mainly involves connexin and pannexin channels and gap junction proteins (35). Exocytotic release of ATP into the extracellular environment has been reported in many cell types, such as neuronal cells (36), platelets (37), lymphocytes (38), mast cells (39), and endothelial cells (40). eATP then acts as a signaling molecule inducing anti- or pro-inflammatory responses depending on its binding to metabotropic P2Y purinergic or ionotropic P2X receptors, respectively, or depending on its concentration (41).

Consider a danger-associated molecular pattern and part of a group of molecules called “alarmins” (42), eATP is involved in recognition of intracellular pathogens and can mobilize an efficient innate immune response (43, 44). This occurs by the secretion of cytokines (45), recruitment of innate immune cells (such as macrophages, neutrophils, eosinophils, and mast cells) (46, 47), and production of nitric oxide (NO) and reactive oxygen species (ROS) (48). eATP also has an influence on specific immune response, since eATP activates T cells that express P2X7 by amplifying T cell receptor (TCR)-induced activation (49), inhibits the differentiation and function of Treg cells (50), and induces the differentiation of Th17 cells (51).

However, high concentrations of ATP and chronic stimulation of the P2X7 receptor also induce T cell apoptosis (52). Interestingly, the P2X7 receptor is expressed on B lymphocytes, and it mediates either cell death or proliferation (53). Wiley et al. showed that in patients with chronic lymphocytic leukemia, apoptosis of lymphocytes was observed upon the activation of the P2X7 receptor. By contrast, with very low eATP concentrations or chronic exposure, the receptor has an anti-apoptotic effect, resulting in an increase in B cell numbers (54). ATP also exerts anti-inflammatory effects *via* P2Y receptors by diminishing the Th1 cell-stimulatory capacity of DCs, by inhibiting lymphocyte effector functions, and by attenuating production by macrophages of pro-inflammatory cytokine (32).

### Adenosine Production by ATP Degradation: Involvement in the Regulation of Immune Responses

To resolve the inflammatory response or to avoid ATP-induced pathological effects, eATP can be dephosphorylated into extracellular adenosine diphosphate and adenosine monophosphate by ectonucleoside triphosphate diphosphohydrolase 1 (or CD39). AMP can be further dephosphorylated to adenosine by 5′-ectonucleotidase (or CD73) (55). Indeed, uncontrolled or chronic inflammation resulting in cell and tissue damage may result from an overactivation of the immune system. Co-regulation between ATP and adenosine is due to purinergic receptors (P1 and P2 receptors for adenosine and ATP, respectively) that are ubiquitously co-expressed. The magnitude of purinergic signaling is controlled by the extracellular nucleotide concentrations that are regulated by the ectoenzymes CD39 and CD73. This purinergic feedback system is confirmed by several studies using ectoenzyme knockout models. Interestingly, CD39^−/−^ and CD73^−/−^ mice are prone during inflammatory conditions to tissue injury such as acute lung injury or intestinal inflammation, highlighting the role of adenosine in terminating the inflammatory response (56, 57). Moreover, increasing the level of adenosine *in vivo* by administration of various adenosine kinase inhibitors downregulates inflammation in different animal models of acute and chronic inflammation (32, 58, 59).

In general, the effects of adenosine on inflammation are opposite to those of ATP, and adenosine acts as an immunoregulatory signal through adenosine 2A receptor binding that inhibits and modulates adaptive and innate immune response functions (60, 61). Adenosine inhibits adhesion to endothelial cells, reduces superoxide anion production by neutrophils, and decreases the secretion of pro-inflammatory cytokines (62, 63) or facilitates the secretion of anti-inflammatory cytokines such as IL-10 (64). Opposite to the action of eATP, adenosine inhibits T cell responses following A2A receptor stimulation, inhibits T cell differentiation and proliferation by blocking TCR signaling and by decreasing IL-2 production (65, 66), reduces cytokine release, induces Treg cells, and inhibits Th17 cells (67, 68).

**Figure 2:**
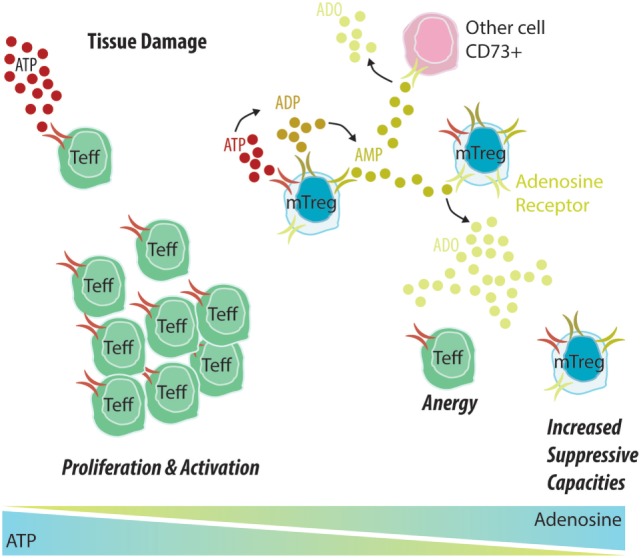
Memory Treg (mTreg) cells induce the degradation of ATP into adenosine, promoting a tolerant environment. (1) An allograft undergoes constant attacks from the immune system, causing tissue damage that leads to the release of ATP into the extracellular environment. (2) In the context of allograft dysfunction, ATP will bind to CD39 expressed by Teff cells, leading to their activation and proliferation and potentiating the inflammatory environment. (3) In tolerant patients, ATP will mostly bind to CD39 expressed by mTreg cells and be further degraded into ADP and AMP by CD39 and into adenosine by CD73. An adenosine-rich environment will promote Teff anergy and increase the suppressive capacity of mTreg cells.

ATP and adenosine thus appear to be crucial endogenous signaling molecules in inflammation and immunity (Figure 2). They may have dual effects on inflammatory responses, depending on the concentration, the receptor used, and the duration of exposure. Therefore, the treatment of immune-related diseases such as graft dysfunction in transplantation may benefit from the control of purinergic signaling molecules.

## Metabolic Regulation of the Immune Humoral Response

Whereas T cell immunometabolism has been a very intense field of investigation over the past few years, the characterization of B cell metabolism is still in its infancy. As is the case with T lymphocytes, the energetic needs of B cells are highly variable, with a necessary transition from the quiescent state to the rapid proliferation phase upon antigen encounter. Naïve and memory B cells as well as long-lived plasma cells persist in the body for years. Upon activation, B cells increase their uptake of glucose (69, 70), and Ab production requires an efficient glycolysis (70). Indeed, provision of the pyruvate dehydrogenase kinase inhibitor dichloroacetate inhibits the glycolysis and suppresses *in vitro* and *in vivo* B cell proliferation and Ab secretion. Mice with B cells deficient in Glut1 exhibit reduced peripheral B cell numbers and total IgM levels in the steady state, and antigen-specific IgM and IgG production fails to increase upon the immunization of Glut1^fl/fl^CD19-Cre mice with NP-OVA (70). Oxidative metabolism or anabolism relies on glucose and glutamine uptake. The relative contributions of glucose and glutamine uptake to ATP generation or to supporting the synthesis of cellular constituents in the different B cell subsets are still unknown, and the use of metabolic trackers will be useful to track the *ex vivo* and *in vivo* fate of carbon donors. Activation of naïve B cells results from a complex integration of signals through BCR, CD40, IL-4R, and TLR (71–73) and causes the engagement of the PI3K/Akt/mTOR pathway. After BCR stimulation, glycolysis is regulated by the PI(3)K signaling pathway (71). IL-4 triggers Glut1 expression, glucose uptake, and glycolysis in splenic B cells (73). BCR activates protein kinase Cβ, which promotes an increase in glycolytic flux (74). The low oxygen tension within the GC light zone sensed by HIF inhibits B cell proliferation, increases their death, and impairs Ab class switching by limiting the expression of activation-induced cytosine deaminase (75).

### Transition From the Quiescent State to Proliferation Requires Metabolic Reprogramming of B Cells

Glycogen synthase kinase 3 (Gsk3) is a metabolic sensor that promotes the survival of naïve circulating B cells. The kinase Gsk3 is ubiquitously expressed in its constitutively active form in nutrient-deprived and resting cells, but its expression rapidly decreases upon phosphorylation after growth factor stimulation (76). Two isoforms (α and β) of Gsk3 exist, and they have highly similar substrate specificities. The proliferation of B cells within the germinal center is associated with the inactivation of Gsk 3β (77). In antigen-driven responses, Gsk3 (α and β) is selectively required for the regulation of mitochondrial biogenesis, glycolysis, B cell size, and production of ROS, in a manner mediated by the costimulatory receptor CD40 and IL-4 (78).

Given the high plasticity of B cells and the continuum of B cell states from immature B cell to plasma cells, the quest to characterize the regulation and the impact of the different metabolic pathways will be challenging but will constitute a fascinating research area.

## Metabolic Regulation of Macrophages

Macrophages are key elements of the innate immune response. Located throughout the body, macrophages maintain tissue homeostasis and behave as immune sentinels. By sensing locally tissue damage and inflammation, macrophages can rapidly modify their functional phenotype to facilitate the elimination of the pathogens and to favor tissue repair. The integration of stimuli from the local environment results in the differentiation of pro-inflammatory macrophages (M1 macrophages) in the presence of pathogen-associated molecular patterns such as LPS (79, 80). M1 macrophages are characterized by their secretion of pro-inflammatory cytokines and antimicrobial properties. By contrast, M2 macrophages differentiate in the presence of IL-4 and IL-13 and are involved in tissue repair and immunoregulatory functions (79, 80). The rewiring of metabolic pathways within macrophages in response to environmental stimuli is a key process for macrophage effector function. The polarizing signals activates canonical signaling pathways known to regulate metabolic processes (1), including the activation of Akt, mTORC1, mTORC2, and AMPK. The differentiation into M1 macrophages relies on aerobic glycolysis as shown by the defect in bacterial killing and myeloid cell infiltration when the metabolic switch to glycolysis is impaired upon HIF-1α deletion (81). M1 polarization is also associated with a defect in mitochondrial function and TCA cycle (82), the latter being truncated at the level of isocitrate dehydrogenase and succinate dehydrogenase (83). The shunt into the TCA participates to the production of itaconate, an important antimicrobial agent inhibiting the bacterial growth such as *Mycobacterium tuberculosis* and *Salmonella enterica* (84). M2 macrophages differs from M1 macrophages not only at their functional level but also at the metabolic processes, oxidative TCA cycle associated with OXPHOS being the major provider of ATP in M2 macrophages. FAO and glutamine metabolism fuels the oxidative TCA cycle in M2 macrophages (83, 85). M2 polarization activates glutamine catabolism and UDP-GlcNAc associated modules (83). Chemokine *Ccl22* production and defect in M2 polarization are observed upon glutamine deprivation (83). Finally, arginine metabolism is strikingly different in M1 and M2 macrophages (86). Production of antimicrobial agent NO is catalyzed by iNOS by converting l-arginine to l-citrulline. By contrast, expression of arginase (ARG-1) by M2 macrophages favors the catabolism of arginine to l-ornithine and urea. This arginase activity limits the production of NO, provides l-ornithine, precursor for the production of l-proline, and favors wound repair function of M2 macrophages through the synthesis of collagen. The alteration of metabolic pathways has been thus proposed as a promising strategy to repolarize macrophages. Macrophages are highly plastic cells that continuously adapt their function to their local environment. Given that most of the investigation on macrophages are performed with isolated cultured cells and singular stimulus, much work remains to be done to understand the adaptation of macrophages to complex *in vivo* environment with multiple stimuli simultaneously.

## Manipulating the Metabolic Pathway in Transplantation

Given that metabolic reprogramming is an essential step to elicit an effector function, it is not surprising that interference with metabolic pathways has been attempted to control the immune response in various preclinical models. Successful attempts to prevent the development of auto- and alloimmune responses have been reported in animal models including tumor vaccination (87), hematopoietic stem cell transplantation (88–91), lupus (92, 93), EAE (94), and heart and skin transplantation (95). Metabolism being a key process shared by all cells within a given individual, one could wonder how to specifically target the metabolism of immune effector cells while preserving low toxicity. Hypothesis-driven experiments in this area rely on the shift to high-metabolic profiles in effector cells. Alloreactive T cells are deleted using a small-molecule inhibitor of the mitochondrial F_1_F_0_ adenosine triphosphate (F_1_F_0_-ATPase) while preserving hematopoietic engraftment and lymphocyte reconstitution in various models of bone marrow transplantation (91). This selective inhibition is based on increased superoxide production, decreased amounts of antioxidants, and hyperpolarization of the mitochondrial membrane potential of alloreactive T cells (91). During the course of GVHD, alloreactive T cells use FAs to support their *in vivo* activation, whereas T cells activated by cellular immunization do not (89). Pharmacological blockade of FAO has thus been shown to prevent GVHD in different models *via* induction of alloreactive T cell apoptosis while sparing the survival of T cells during normal immune reconstitution (89). The antidiabetic drug metformin has been used to target alloreactive T cells in GVHD (88, 90, 91), organ transplantation (95), and lupus (92). Disease progression and a CD4 T cell-skewed response in lupus-prone mice are reverted and controlled by combining 2-DG and metformin (92). Despite the treatment of lupus-prone mice with 2-DG and metformin for 3 months, the authors do not report any physiological side effects of such a long-term treatment. The absence of toxicity to normal tissues may seem surprising. A therapeutic window could exist for drugs that target metabolic treatment, as also demonstrated by the wide use of metformin to treat type 2 diabetes.

Examples of control of alloimmune response in models of organ transplantation are sparse. Combinatory treatment with 2-DG, DON, and metformin prevents or delays graft rejection in fully mismatched heart or skin allograft transplantation models (95). A better characterization of the metabolic requirement of allogeneic T cells during the course of allogeneic response is needed to design innovative treatments that could target immune cells that escape from standard immunosuppressive regimens. An increase of effector memory re-expressing CD45RA CD8 T cells (TEMRA CD8) is associated with an increased risk of kidney dysfunction in kidney transplant (KT) recipients (96). We have recently demonstrated that IL-15 activate TEMRA CD8 cells from KT recipients, despite immunosuppressive therapies, and promote endothelial inflammation as shown by the upregulation of CX3CL1 in human umbilical vein endothelial cells in an IFN-γ- and TNF-α-dependent manner (97). TEMRA CD8 cells exhibit an active metabolic state characterized by a large pool of pre-formed ATP and high expression of genes involved in glycolysis and the PPP. TEMRA CD8 adapts their metabolism to stimulation by increasing their ECAR and oxygen consumption rate, demonstrating thereby their metabolic fitness. Finally, interfering with the processes of glycolysis and glutaminolysis in TEMRA CD8 cells from KT patients efficiently prevents the endothelial inflammation (97).

In the field of solid organ transplantation, eATP may be particularly important, since it participates in the fine modulation of the immune response, acting as a danger signal that will induce a pro-inflammatory environment. Inflammation is dampened upon ATP degradation, under the controlled expression of CD39 and CD73 (32). In particular, the expression of CD39 by APC or regulatory T cells appears to be of crucial importance in their immunomodulatory functions (98, 99). Moreover, CD39 expression is a fundamental determinant of human Treg function and stability (100), and recent studies have shown that CD39 expression is restricted to the most stable and suppressive subset of CD4 Treg cells (101), the human memory Treg (mTreg) cells, and show immune suppression through the production of adenosine (102, 103).

In the particular state of tolerance in kidney transplantation, we reported an increased proportion of mTreg cells in tolerant patients and not in patients with stable graft functions (104). Interestingly, mTreg cells from patients with stable graft function were unable to degrade eATP, whereas this ability was preserved in mTreg cells in tolerant patients (105). This lack of degradation capacity was not due to immunosuppressive treatments. Finally, a reduced mTreg and mTeff cells expressing CD39 was observed in patients with acute cellular rejection, and mTreg cells in transplant patients with stable graft function displayed more potent suppressive capacity than those of non-immunosuppressed controls (106). Thus, the authors of those findings propose that determining changes within these T cell subsets could help identify patients at risk of renal allograft rejection and, furthermore, that they could be considered for clinical purposes due to their suppressive properties.

The depletion of eATP through its degradation into adenosine is now considered an immunomodulatory mechanism (100, 105, 107). Adenosine 2A receptor signaling attenuates kidney graft rejection and alloantigen recognition and promotes peripheral tolerance by inducing the generation of Treg cells and the anergy of conventional T cells (108–110). There is mounting evidence that the inflammatory response that accompanies rejection and chronic allograft dysfunction involves purinergic signaling. Roberts et al. report that the CD39, CD73, and A2 signaling pathways attenuate cardiac, liver, and lung ischemia–reperfusion injuries and reduce lung and kidney allograft dysfunction (111). Current studies are investigating the potential for A2 receptor agonists or molecules targeting the purinergic pathway to attenuate alloantigen recognition and transplant rejection (108, 112–114).

Given that metabolic reprogramming is an essential step to elicit an effector function, it is not surprising that interference with metabolic pathways has been attempted to control the immune response.

## Concluding Remarks

This review clearly shows the cross talk between metabolism and cell-based immunity, with glycolysis, OXPHOS, and lipid metabolism playing instrumental roles in T cell response by acting on T cell motility and cytokine secretion but also regulating this response by way of eATP and adenosine production. Metabolism is also of importance in the humoral response, particularly through regulating the transition of B cells from the quiescent state to proliferation but also through interacting directly with their regulation and/or suppressive properties. The effects of current immunosuppressors on metabolism and the manipulation of metabolic pathways in transplantation thus appear to be instrumental and should clearly be taken into account in the design of future therapies.

## Author Contributions

All authors listed have made a substantial, direct, and intellectual contribution to the work and approved it for publication.

## Conflict of Interest Statement

The authors declare that the research was conducted in the absence of any commercial or financial relationships that could be construed as a potential conflict of interest.
